# The feasibility of axial and coronal combined imaging using multi-detector row computed tomography for the diagnosis and treatment of a primary spontaneous pneumothorax

**DOI:** 10.1186/1749-8090-6-71

**Published:** 2011-05-14

**Authors:** Do Hyung Kim

**Affiliations:** 1 Department of Thoracic & Cardiovascular Surgery, Pusan National University, Yangsan Hospital, Yangsan, Korea

## Abstract

**Background:**

The preoperative detection of emphysema like changes (ELCs) is necessary for the successful treatment of pneumothorax. High resolution computed tomography (HRCT) has been used for the preoperative detection of ELCs. However, the traditional HRCT method uses only the axial view, which is perpendicular to the distribution of ELCs. This is not an ideal diagnostic method for the evaluation of ELCs.

**Methods:**

Forty-eight patients with pneumothorax had multi-detector computed tomography (MDCT) reconstruction using the coronal view. ELCs were evaluated in the axial and coronal view by a radiologist. A surgeon performed intra-operative examinations of the ELCs. The sensitivity of the different views was compared.

**Results:**

The detection sensitivity was 74.4% (70/94) for the axial view and 91.5% (86/94) for the axial-coronal combined view. The intra-operative detection rate was 95.7% (90/94). The preoperative detection of ELCs on the axial-coronal combined view was significantly higher than on the conventional axial view alone (p < 0.01).

**Conclusions:**

Evaluation of ELCs on the axial and coronal combined HRCT improved the sensitivity of preoperative detection of ELCs compared to the conventional single axial HRCT. This increased sensitivity will help decrease the recurrence with VATS.

## Background

The recurrence of a pneumothorax after video assisted thoracic surgery (VATS), after the treatment of primary spontaneous pneumothorax, is higher than after thoracotomy procedures [1-8]. Although it is difficult to prove the cause of higher recurrence rates, it has been suggested that videoscopic inspection is less accurate than direct inspection; this is because the lung is collapsed during VATS, and therefore the frequency of overlooking emphysematous like changes (ELCs) is higher with VATS procedures.

The multi-detector computed tomography (MDCT) provides extended volume coverage of the longitudinal axis and high image quality in a short time, using an even higher pitch. With volumetric CT acquisition, thin collimation, and high pitch, and contiguous thin slice images can be generated that facilitate accurate assessment of focal and diffuse lung disease [9]. The volumetric CT acquisition also makes it possible to generate high-resolution multi-planar reformation (MPR) images. These technical advances have also improved the agreement between clinicians in the diagnosis of emphysematous lung disease, when the MPR images are used in conjunction with standard axial imaging. Furthermore, structures adjacent to the chest wall, especially those close to the apex and diaphragm, can be visualized more clearly on the MPR images than on the axial images [10].

Use of the MPR images for the diagnosis of pneumothorax might aid in the preoperative detection and evaluation of ELCs. The purpose of this study was to evaluate the efficacy of axial and coronal combined views using the MDCT compared to conventional axial views, for the initial treatment of primary spontaneous pneumothorax.

## Methods

Sixty-nine patients with primary spontaneous pneumothorax were admitted to the hospital. All patients underwent 16 channel MDCT scanning (Somatom Sensation 16, Siemens Medical Systems, Erlangen, Germany) to evaluate the number, location, and type of ELCs. Twenty-one patients that had no ELCs detected on the MDCT were excluded as well as those with a history of surgery for a previous pneumothorax. Forty-eight patients were finally enrolled in this sturdy. All participating patients provided informed consent. There were 45 men and 6 women (age range, 14-42 years; mean, 22.9 ± 8.4 years).

### Axial and coronal HRCT protocol

The imaging parameters were as follows: 1.0 mm collimation, 120 kVp, 200 mA, 0.5 sec gantry rotation time and a table speed of 15 mm per rotation. All patients were scanned in the cranial to caudal direction from the lung apex to the lung base. The patients were instructed to maintain suspended inspiration during CT acquisition. From each acquisition, two sets of lung images were systematically reconstructed: 1 mm thick axial CT scans and 1 mm thick coronal images. The axial images of the upper and lower lung fields were obtained at 1 mm and 10 mm intervals and reconstructed using a high spatial frequency algorithm.

### Interpretation of CT images with regard to ELCs

ELCs were defined as the presence of single or multiple cystic lung lesions more than 5 mm in size, and multiple conglomerated cysts identified as a single lesion. All images were displayed using a picture archiving and communication system (PACS) work station (M-view, Marotec Inc, Seoul). One board-certified chest radiologist first assessed the axial CT and marked the ELCs on the CT image. The same procedure was used for the coronal view, to obtain independent information on each view. After each view was evaluated, we re-evaluated to obtain more exact information on both views. The combined axial-coronal view was defined as coronal view added axial view.

Before surgery, one thoracic surgeon analyzed the number, location, and type of ELCs according to the MDCT data. After analysis of the ELCs, a thoracic surgeon performed an axillary thoracotomy for direct inspection of the ELCs.

The operation and inspection of ELCs was performed as follows: After inducing general anaesthesia with a double lumen intubation, the patient was placed in the lateral position on the operating table with the ipsilateral arm flexed and abducted 90°. The axillary thoracotomy was performed at the 3rd intercostals space for the direct inspection. Lung inflation was sustained for a precise inspection of ELCs. The ELCs, which were marked in the axial and coronal CT, were inspected through the thoracotomy. Repeated inspections considering both views were performed to identify the ELCs not identified by CT. After examining the ELCs, a wedge resection containing the enough resection margin of ELCs was performed. The range of the resection was determined according to the coronal CT findings and gross findings during surgery. In cases where an ELC was detected on the CT but not in the surgical field, the suspected area was resected. The final confirmation of ELCs was based on the pathology reports.

### Data analysis

The ELCs that were preoperatively detected were compared to the visual inspection during surgery and the pathology reports. A true negative could not be determined because CT negative patients were not enrolled in this study. The true positives, false positives and false negatives could be determined. The sensitivity and positive predictive values were defined.

Values are expressed as means ± standard deviation (SD). The number of preoperatively detected ELCs was compared between each view and the results analyzed by the paired t-test. The sensitivity of the axial CT, coronal CT, and combined views CT were examined by McNemar's test. A P value < 0.05 was considered statistically significant. Data were analyzed with SPSS 11.0 software (SPSS Inc, Chicago, IL, USA).

## Results

All patients had bullectomy performed through an axillary thoracotomy. The mean operation time was 24.5 ± 7.5 minutes (range, 15-34 minutes). There was no associated morbidity or mortality. The mean duration of chest tube drainage after surgery was 3.2 ± 1.9 days (range, 2-9 days). The mean hospital stay after the operation was 4.5 ± 1.9 days (range, 3-10 days). The mean number of ELCs detected was 1.4 ± 1.0 (range, 1-4), 1.5 ± 1.0 (range, 1-4) and 1.7 ± 1.0 (range, 1-5) in the axial, coronal, and axial and coronal combined views (Table 1). Although the detection with the coronal view was not higher than the axial view (p = 0.137), the combined axial-coronal view had a significantly higher detection rate than the conventional axial view (p < 0.01).

**Table 1:** Patients characteristics

Patients characteristics	
Mean age	22.9 ± 8.4 years (range, 14-48)
Male : Female	42:6
Right: Left	31: 17
Operation time	24.5 ± 7.5 minutes (range, 15-34 minutes)
Mean number of ELC*	
Axial view	1.4 ± 1.0 (range: 1-4)
Coronal view	1.5 ± 1.0 (range, 1-4)
Axial& coronal view	1.7 ± 1.0 (range, 1-5)

A total of 94 ELCs were pathologically confirmed. Ninety ELC's were grossly visualized and pathologically confirmed and four ELC's were confirmed only by resection of suspicious areas. Eighty eight ELCs were located in the apex of the upper lobe, 6 ELCs were located in the superior segment of the lower lobe. Eighty-seven ELCs were detected by the combined axial-coronal view. Sixty-three ELCs were detected in the axial and coronal views, 18 ELCs in the coronal view, and six ELCs in only the axial view. There were no false positive ELCs in this study; hence the positive predictive value was 100% in all views. The sensitivity of the axial, coronal, and combined CT views, as well as direct inspection were: 73.4% (69/94), 84.0% (79/94), 92.6% (87/94), and 95.7%(90/94), respectively. There was no significant difference in the sensitivity between axial and coronal views (p = 0.125). However, the sensitivity of the axial-coronal combined view was higher than the axial or coronal CT views (p < 0.001, = 0.003). When the sensitivity of the combined axial-coronal views was compared to direct inspection during surgery, there was no significant difference noted (p = 0.388, Table 2).

**Table 2:** Sensitivity of axial, coronal, combined, and direct inspection

Sensitivity	
Axial view	70/94(74.4%)
Coronal view	79/94(84.0%)
Combined view	86/94(91.5%)
Direct inspection	90/94(95.7%)

*The number of coronal view is not significantly higher than axial view (p = 0.137), but combined axial-coronal view combined view is significantly higher than conventional axial view (p < 0.01).

## Discussion

ELCs were confirmed in 85% of the patients undergoing thoracotomy. The number of ELCs in the affected lung is significantly greater in patients with a history of recurrent pneumothorax and in patients that need a thoracotomy [11,12]. Although the exact mechanism of recurrence is not known, the presence of identifiable ELCs may be the most common cause of recurrent pneumothorax [13]. Therefore, the resection of as many ELCs as possible will likely reduce the recurrence rate of pneumothorax.

HRCT imaging of the chest was developed to allow for improved diagnostic accuracy, sensitivity, and specificity for the evaluation of the pathology of lung parenchymal disease. The thinner collimation of HRCT results in marked improvement in spatial resolution compared to conventional CT. The HRCT has been used to identify ELCs in patients with primary pneumothorax. Identification of ELCs may be an indication for surgery at some centers. Yim et al [14] reported that 53.6% patients had blebs or bullae in the contralateral lung. During the follow-up period, 26.7% patients with contra-lateral blebs developed pneumothorax in the untreated lung. CT scanning can be used to predict the risk for recurrence in such cases. Kim et al [15] proposed that ELCs identified by HRCT was a good indication for surgery and that the HRCT shortened the observation time of ELC recurrences. The HRCT is clinically useful for the diagnosis of pneumothorax.

However, the conventional HRCT does not provide imaging of intervals less than10 mm. Therefore, the images cover only approximately one tenth of the entire lung field. The scanning time would be unacceptably long to obtain contiguous thin images for all lung fields. Thus, ELCs within the 10 mm intervals would not be detected. Therefore, the HRCT was not widely used for the diagnosis of pneumothorax. However, the MDCT is capable of imaging at full resolution and improved on the limitations of the HRCT. Imaging of all lung fields could be performed quickly and the images reconstructed retrospectively. Alternatively, the scanner could be configured to perform contiguous 1 mm sections for an HRCT examination and evaluate cystic lung lesions less than 10 mm in size.

However, most ELCs are located at the apex of the lungs and cranio-caudally distributed along the bronchial trees. Therefore cranio-caudal evaluation is necessary for accurate examination of ELCs. HRCT is traditionally performed by axial imaging. Although axial imaging has the advantage of the central and peripheral areas being observed simultaneously, it is perpendicular to the cranio-caudal observations [16,17]. A new directional image might be necessary to overcome the disadvantages of traditional imaging methods. The introduction of coronal imaging might provide more accurate examination of ELCs in cases of primary spontaneous pneumothorax.

Recently, computed tomography (CT) technology has improved with the advent of the multidetector row technique and the introduction of the spiral CT. The MDCT permits reconstruction of coronal images. There has been a growing trend and interest in using coronal images for the evaluation of thoracic abnormalities, including pulmonary emboli, focal parenchymal diseases, diffuse lung diseases, and bronchiectasis. The coronal image reconstruction has made radiological diagnoses more accurate and has provided more useful information for surgical preparation [18]. However, initially coronal images were not performed due to the additional cost. This problem has been recently resolved by the introduction of PACS; and the use of coronal images is now more feasible.

The coronal image has some radiological advantages compared to the axial image. The number of images used for coronal multi-planar reconstruction (MPR) views of the whole lung, on the thin-section CT, is only about one-half to one-third of the original transverse whole lung thin-section CT images; this makes imaging of the whole lung feasible, even if the same slice thickness is used for both coronal MPR views and whole lung thin-section CT images. In the cases with primary pneumothorax, more than 150 axial images are necessary to evaluate ELCs from the apex to the hilar regions of the lung. However, less than one-third of the images are necessary using the coronal view, and the image quality is better [19].

The addition of coronal imaging of ELCs increases the detection rate of ELCs. The coronal images can be used to reassess ELCs in cases where the diagnosis of ELCs is difficult or vague on axial images, and for the identification of new ELCs not observed on axial imaging. In this study, there was no significant difference in the sensitivity of axial and coronal images; when the data was analyzed, the coronal view provided additional value. Seventeen percent of all ELCs were newly detected on the coronal view. In addition, the sensitivity was increased when the axial and coronal views were combined compared to the traditional axial HRCT. Although seven ELCs were overlooked in the combined view compared to direct inspection, the sensitivity of the combined view was similar to direct inspection. The independent use of the coronal view is not recommended for the diagnosis of pneumothorax; however, the combined evaluation improves on the sensitivity of detection of ELCs.

Moreover, the coronal view has additional clinical advantages compared to the axial images. A new growing bulla at the staple line is another cause of recurrent pneumothorax; such lesions are due to incomplete resection of the cystic parenchymal lesions. Therefore, the evaluation of the relationship between normal lung and intra-parenchymal cystic pathology is necessary for the complete resection of ELCs [20].

**Figure 1:**
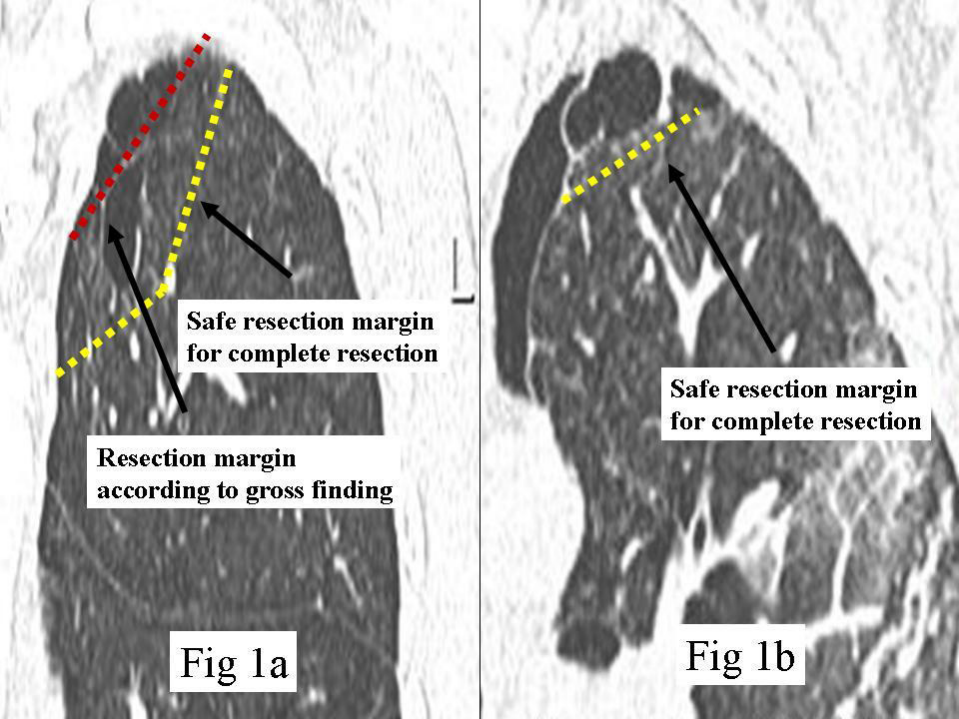
**The ideal resection margin of bleb and bullae**. The dotted line shows the ideal resection margin of ELC in the treatment of pneumothroax. The visible lesions which contain intraparenchymal cystic lesions are a part of ELC such as tip of ice burg. The wider resection of lung will be necessary for complete resection (figure 1a). On the other hand, the resection of visible lesion is enough in case that there was no intraparenchymal pathologic lesion (figure 1b).

The intra-parenchymal cystic pathology aids in determining the extent of the lung resection. For example, a wider resection of the lung is necessary for a complete resection if there is an intraparenchymal cystic lesion; such lesions cannot be detected on gross inspection. If only those ELCs that are visible on gross inspection are resected, the intraparenchymal cystic lesions will be missed, and the chance of an incomplete resection increased (figure 1a, figure 1b).

Furthermore, the assessment of the lung apex using axial images on HRCT is difficult; this is because the area of lung apex is limited and very small and many cuts are necessary to discriminate between emphysematous lesions and normal parenchyma in the apex of the lung. However, the coronal view can show the relationship between normal lung parenchyma and intra-parenchymal cystic lesions using only one or two images. Therefore, the coronal view is more useful for deciding on the extent of lung resection than the axial view, and might reduce the frequency of new growing bullae.

## Conclusions

The combined axial-coronal HRCT increased the sensitivity of the preoperative detection of ELCs. Coronal imaging alone was not significantly better than axial imaging alone. The coronal imaging helped with decisions on the extent of the resection. The combined axial-coronal view was a more effective clinical tool for preoperative diagnosis and surgical planning than simple axial HRCT for the diagnosis and treatment of primary spontaneous pneumothorax

## List of abbreviations

MDCT: multi-detector computed tomography; ELC: emphysema like change; HRCT: high resolution computed tomography; VATS: video assisted thoracic surgery; PACS: picture archiving and communication system; MPR: multi-planar reformation

## Competing interests

The author declares that they have no competing interests.

## Authors' contributions

DHK carried out the clinical work, drafted the manuscript and participated in its design. Author read and approved the final manuscript.
